# Real-time detection of aging status of methylammonium lead iodide perovskite thin films by using terahertz time-domain spectroscopy

**DOI:** 10.1007/s12200-024-00128-0

**Published:** 2024-07-29

**Authors:** Jinzhuo Xu, Yinghui Wu, Shuting Fan, Xudong Liu, Zhen Yin, Youpeng Yang, Renheng Wang, Zhengfang Qian, Yiwen Sun

**Affiliations:** 1 Department of Biomedical Engineering, School of Medicine, Shenzhen University, Shenzhen, 518060 China; 2 Guangdong Provincial Key Laboratory of Durability for Ocean Civil Engineering, College of Civil and Transportation Engineering, Shenzhen University, Shenzhen, 518060 China; 3 Key Laboratory of Optoelectronic Devices and Systems of Ministry of Education and Guangdong Province, College of Physics and Optoelectronic Engineering, Shenzhen University, Shenzhen, 518060 China

**Keywords:** Perovskite, Terahertz spectroscopy, Ageing, Real-time detection

## Abstract

The inadequate stability of organic–inorganic hybrid perovskites remains a significant barrier to their widespread commercial application in optoelectronic devices. Aging phenomena profoundly affect the optoelectronic performance of perovskite-based devices. In addition to enhancing perovskite stability, the real-time detection of aging status, aimed at monitoring the aging progression, holds paramount importance for both fundamental research and the commercialization of organic–inorganic hybrid perovskites. In this study, the aging status of perovskite was real-time investigated by using terahertz time-domain spectroscopy. Our analysis consistently revealed a gradual decline in the intensity of the absorption peak at 0.968 THz with increasing perovskite aging. Furthermore, a systematic discussion was conducted on the variations in intensity and position of the terahertz absorption peaks as the perovskite aged. These findings facilitate the real-time assessment of perovskite aging, providing a promising method to expedite the commercialization of perovskite-based optoelectronic devices.

**Graphical Abstract:**

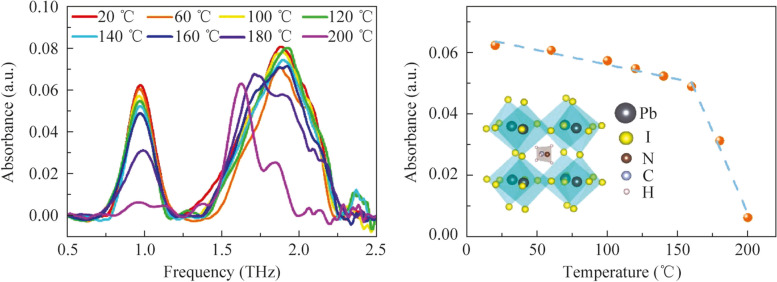

## Introduction

Organic–inorganic hybrid perovskites with a chemical formula of ABX_3_ have emerged as promising semiconductors for optoelectronic applications owing to their remarkable characteristics, which include high charge carrier mobility, large optical absorption coefficient, broad response spectrum, facile synthesis process, and cost-effectiveness [1–4]. These advantageous features render them highly suitable for a variety of fields such as solar cells [5, 6], biosensors [7–9], photodetectors [10, 11], and light-emitting diodes [12, 13]. Considerable progress has been made in the realm of perovskite-based optoelectronic devices, exemplified by the substantial enhancement of perovskite solar cell power conversion efficiency from 3.8% to 25.86%, surpassing that of single crystal silicon [14, 15]. Moreover, photodetectors utilizing perovskite materials have achieved notable photoresponsivity comparable to commercial detectors [16], while perovskite LEDs have demonstrated exceptional attributes encompassing color rendering, brightness, and luminous efficiency [17, 18].

Despite their immense potential, the commercial deployment of perovskites in optoelectronic devices encounters certain obstacles. The stringent requirements of perovskite synthesis conditions and the instability of their encapsulation devices pose substantial challenges to their extensive practical applications [19–21]. Even minor alterations in doping elements, impurities, or synthesis conditions can profoundly impact the crystal structure, lattice distortion, and defect formation of perovskites, thereby affecting their stability and reproducibility of optoelectronic properties. For instance, the doping of Cs^+^ ions into FAPbI_3_ (FA = CH(NH_2_)_2_) perovskite is a common approach aimed at improving its photoresponse performances and enhancing stability in atmospheric conditions. However, different research teams have obtained varied optimal results. Lee et al. [22] reported that substituting 10% of FA^+^ ions with Cs^+^ ions yielded the best outcome, while Li and the co-authors [23] achieved the best results by substituting 15% of FA^+^ ions. On the other hand, Yi et al. [24] achieved optimal performance by replacing 20% of FA^+^ ions with Cs^+^ ions. These discrepancies could potentially be attributed to minor differences in synthesis conditions, such as heating temperature, heating duration, and substrate choice. Additionally, upon integration into optoelectronic devices, environmental factors such as oxygen, moisture, heat, and ultraviolet light can induce perovskite degradation, resulting in detrimental effects on their optoelectronic performance and long-term stability [25–27]. Zhao et al. [28] fabricated a perovskite photodetector, which exhibited a remarkable decline of 98% in photocurrent after a 24 h aging testing. Despite their implementation of encapsulation techniques to enhance the device stability, the photoresponse of the detector still experienced a substantial degradation of 27% after undergoing a 168 h aging process. Additionally, Lu et al. [25] synthesized a flexible perovskite photodetector that demonstrated excellent light response characteristics, encompassing a broadband response spectrum from the UV to the NIR, as well as effective low-light detection, with a photocurrent reaching 2.26 nA at an extreme low incident light intensity of 9.36 × 10^−4^ mW/cm^2^. Nevertheless, following an 80 min aging, the photodetector experienced a decline of 25% in photocurrent. Consequently, real-time characterization of perovskite films both in the synthesis process and after encapsulation for devices application, enables the monitoring of the synthesis quality and the aging status of perovskite optoelectronic devices, assumes crucial significance for the fundamental research of perovskites and their commercial applications.

Detection techniques such as scanning electron microscopy (SEM), photoluminescence (PL), Raman spectroscopy, and X-ray diffraction (XRD) are commonly utilized in the synthesis processes of perovskites to evaluate their physical properties, structural features, and crystal quality, thereby ensuring the production of high-quality perovskite materials. However, assessing the aging behavior of perovskite optoelectronic devices presents challenges due to device packaging requirements and the necessity for non-destructive testing. While thermal decomposition tests are frequently employed to study perovskite stability, this method lacks the capability for real-time, in situ, and non-destructive detection [29]. Hence, it has become crucial to develop technologies that enable real-time and non-destructive detection of perovskite materials capable of penetrating the encapsulation layers.

In recent years, terahertz (THz) technologies have been extensively applied in varies fields including industrial defect detection, biological sensing, material characterization, and security inspection owing to their advantages of non-destructive and sensitivity to material composition and structure [30–37]. Moreover, terahertz waves exhibit strong penetration capabilities in non-polar dielectric materials such as quartz, sapphire, and plastics, which makes them a powerful tool for in situ detection of the encapsulated devices. Moreover, terahertz technology has significantly contributed to the study of perovskite materials, including methylammonium lead halide perovskite (MAPbX_3_ (X = I, Br, Cl)). Numerous studies have reported on the terahertz spectra of these perovskites [33, 35–39]. For example, La–o-vorakiat et al. investigated the terahertz spectra of MAPbI_3_ perovskite and identified two phonon modes around 1 and 2 THz, attributed to the buckling of the I-Pb-I bonds and the stretching of the Pb-I bonds, respectively [38]. Similarly, Sendner et al. conducted quantitative measurements of the lattice vibrations of MAPbX_3_ (X = I, Br, Cl) at room temperature, identifying phonon modes in the vicinity of 1 and 2 THz [39]. They also estimated the upper limit of the charge carrier mobility in MAPbI_3_ to be 200 cm^2^/(V⋅S) based on these measurements. It is foreseeable that with the aging of perovskite, defects will emerge in its crystal structure, leading to the occurrence of defects, dislocations, and even disintegration in the framework composed of metal and halogen. This will inevitably result in changes in phonon vibration modes, potentially providing opportunities for terahertz spectroscopy to detect the aging of perovskites. However, it is worth noting that there have been no reports utilizing terahertz detection technology to real-time monitor the aging of perovskite materials.

In this paper, MAPbI_3_, a widely used organic–inorganic hybrid perovskite with outstanding photoresponse performance, was selected as the research subject. Different aging levels of perovskite thin films obtained through thermal decomposition and natural aging methods were characterized using a terahertz time-domain spectroscopy system. The study revealed the presence of two distinct terahertz absorption peaks in the perovskite thin film, located at 0.968 and 1.895 THz, respectively. The intensity of the terahertz absorption peak at 0.968 THz exhibited a monotonic decay with increasing aging level for both aging methods employed in the perovskite samples. By real-time detection of the intensity of this absorption peak, the degradation status of the perovskite could be quantitatively analyzed. These findings may offer new insights into the real-time monitoring of perovskite aging status and performance calibration of perovskite optoelectronic devices.

## Materials and methods

### Preparation of the aged MAPbI_3_ films with varies aging levels

Anhydrous dimethylformamide (DMF), dimethyl sulfoxide (DMSO) and chlorobenzene were acquired from Alfa Aesar. Methanaminium iodide (MAI) and lead(II) iodide (99.99%) were procured from Xi’An Baolaite Photo-Electric Technology Co., Ltd., China.

The experiment was conducted inside a glove box. A precursor solution was prepared by combining stoichiometric amounts of PbI_2_ and MAI (1:1) in a mixed solvent of DMF and DMSO (9:1 v/v). The solution was then spin-coated onto the oxygen plasma treated hydrophilic sapphire substrates at a speed of 4000 r/min for 35 s, with the addition of chlorobenzene within the first 15 s to quench the reaction. The resulting MAPbI_3_ films were annealed at 100 °C for 15 min. To induce varying aging levels of perovskite films under different aging conditions, two methods for the aging process: thermal decomposition and ambient temperature natural aging were employed in this work. In the thermal decomposition aging method, the perovskite films were exposed to ambient air and heated at temperatures ranging from 60 °C to 200 °C with a heating duration of 5 min for different samples. Subsequently, the samples were naturally cooled down to room temperature for subsequent characterization. In the ambient room temperature natural aging method, the perovskite films were placed in the atmosphere environment with a temperature of 20 °C and a humidity of 43% for natural aging. Characterization of the samples was conducted weekly, and the aging process lasted for 7 weeks.

### Characterization

The morphology of the MAPbI_3_ thin films prepared in this study was characterized using scanning electron microscopy (SEM) at an accelerating voltage of 15 kV (Jeol-7500F). The crystalline structure of the films was examined by X-ray diffraction (XRD) using a Rigaku KYOWAGL AS-XA H-12 instrument, with Cu K_*α*_ radiation (*λ* = 1.54178 Å) monochromatized by a graphite monochromator. The UV–vis absorption spectra was measured by a Shimadzu UV 2550 spectrophotometer equipped with an integrating sphere over the spectral range 500–800 nm. Room temperature photoluminescence (PL) and time-resolved PL spectra were acquired using a fluorescence spectrophotometer (FS5, Edinburgh Instruments Livingston, UK). For terahertz frequency detection, we employed a terahertz time-domain spectroscopy (TeraPulse 4000, TeraView, UK) system, which utilizes an 800 nm femtosecond fiber laser with a pulse duration of 100 fs, a repetition rate of 80 MHz, and a laser power of 800 mW. Photoconductive antenna is designed for use as either an emitter or a receiver of terahertz radiation in the frequency region from 0.1 to 4.5 THz. However, the detectable frequency range is 0.5–2.5 THz in this work because of the scanning and thin-film absorption characteristics of the perovskite material. To prevent moisture interference and ensure sample stability during testing, dry nitrogen was continuously supplied to the chamber throughout the experiment.

## Results and discussion

**Fig. 1:**
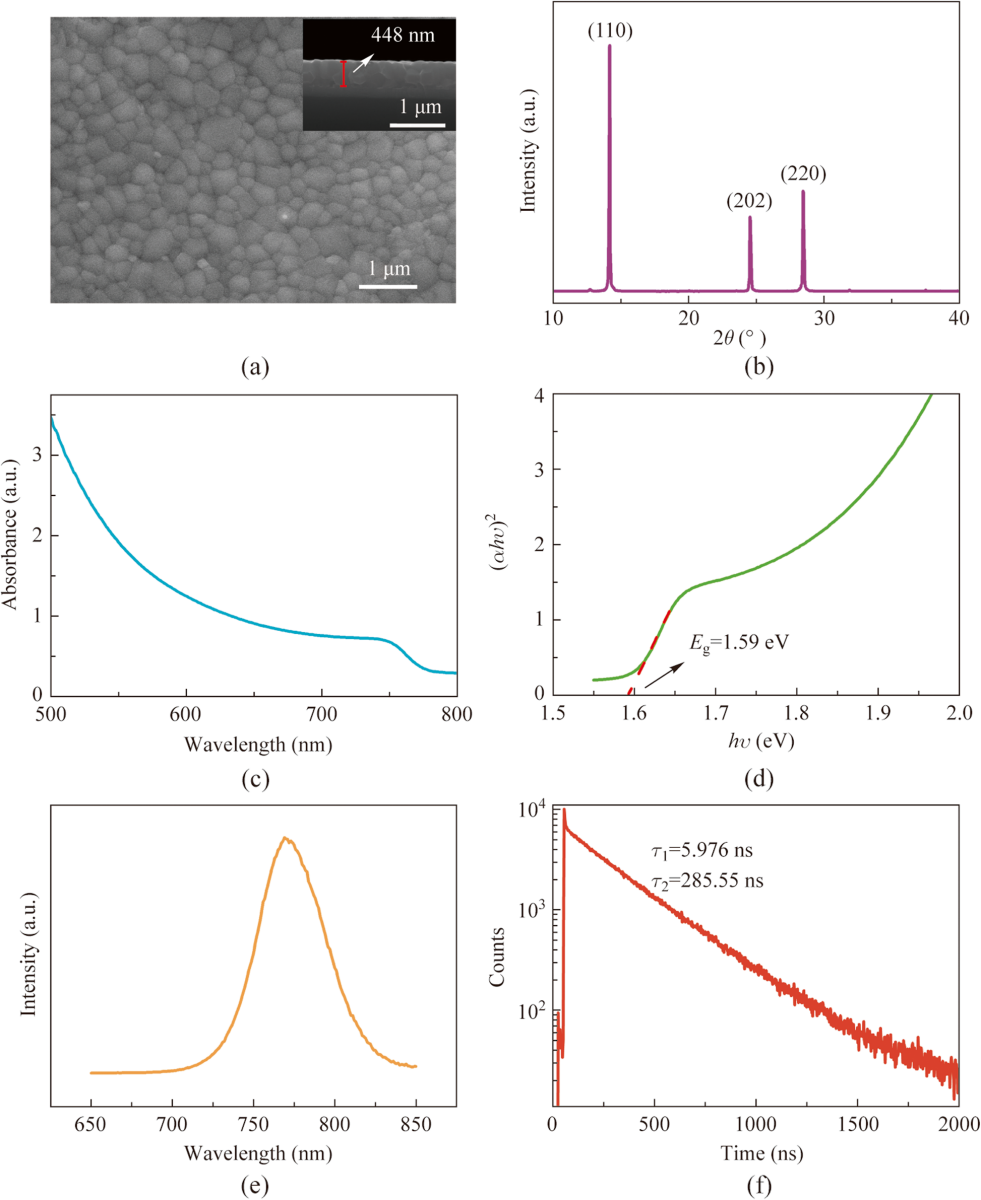
**a** SEM images, **b** XRD spectrum, **c** UV–vis spectrum and **d** the corresponding *hν*–(*αhν*)^2^ curve, **e** PL and **f** TRPL of the as-prepared MAPbI_3_ films

To comprehensively analyze their composition and quality, the as-prepared perovskite films were systematically characterized. The surface morphology of the perovskite film was characterized using SEM, as shown in Fig. 1a. The SEM image reveals a smooth surface with homogeneous grain distribution, indicating the formation of a dense, crack-free film of high quality. Additionally, the cross-sectional SEM analysis, as illustrated in the inset of Fig. 1a, yielded a measured thickness of 448 nm for the perovskite film. To obtain additional information regarding the local crystal structure, the XRD analysis was conducted on the MAPbI_3_ films. The resulting diffraction pattern, shown in Fig. 1b, exhibits a well-defined diffraction pattern corresponding to the tetragonal phase, indicating excellent crystallinity of the as-prepared MAPbI_3_. The diffraction peaks observed at 14.10°, 24.87°, and 28.42° (2*θ*), corresponding to the (110), (202), and (220) reflections of the perovskite structure, respectively, confirm the presence of the desired crystalline phase in the as-prepared organic–inorganic hybrid perovskite sample [40, 41]. The remarkably narrow full width at half maximum (FWHM) of these characteristic peaks demonstrates the exceptional crystallinity and structural homogeneity of the MAPbI_3_ film. To quantitatively estimate the grain size of the MAPbI_3_ films, we utilized the Scherrer equation [41]:

$$D=\frac{K\cdot \lambda }{\beta \cdot \text{cos} \ \theta },$$

where *D* represents the grain size, *K* is the shape factor (typically taken as 0.89), *λ* is the wavelength of the X-rays, *β* is the full width at half maximum (FWHM) of the diffraction peak, and *θ* is the diffraction angle. In our measurements, the X-ray wavelength is 1.54178 Å, the FWHM is 0.1°, and the diffraction angle is 7.05°. Using these parameters, the grain size of the MAPbI_3_ films was calculated to be approximately 79.18 nm. Furthermore, the absence of any other extraneous peaks further indicates the absence of impurities or unwanted phases, reaffirming the high purity and superior quality of the synthesized perovskite. These findings underscore the successful fabrication of a well-defined and pristine perovskite structure.

The UV–vis absorption analysis of MAPbI_3_ films was conducted to investigate its optical properties. The obtained UV–vis absorption spectrum shown in Fig. 1c exhibits a prominent absorption edge in the visible range, indicative of a high absorption coefficient within the range of 500 to 800 nm for the as-prepared perovskite films. According to the Tauc plot method [36], the bandgap width (*E*_g_) of a semiconductor can be calculated from its UV–vis absorption curve by using the following equation:

$${\left(\alpha hv\right)}^\frac{1}{n}=A\left(hv-{E}_{\text{g}}\right),$$

where *α* is the absorption index, *ν* is the frequency of light, *h* is the Planck constant, and *A* is a constant. The exponent “*n*” varies with the semiconductor type: 1/2 for direct bandgap and 2 for indirect bandgap semiconductors. For calculate the bandgap of MAPbI_3_, the *hν* − (*αhν*)^2^ curve was plotted and shown in Fig. 1d. According to the above equation, the intersection of the reverse extrapolation curve’s tangent line with the *x*-axis at *y* = 0 corresponds to the bandgap width of perovskite. Consequently, the bandgap of MAPbI_3_ is approximately 1.59 eV, slightly higher than the previously reported value of 1.55 eV, possibly due to the presence of a small amount of PbI_2_ in the perovskite. The optical characteristics and carrier complex behavior of the perovskite films were assessed through photoluminescence (PL) and time-resolved photoluminescence (TRPL) tests. In the PL spectrum obtained with 532 nm excitation in Fig. 1e, a PL peak centered at 769 nm was detected, exhibiting a slight red shift compared to previous results [42]. This phenomenon is attributed to the Urbach band tailing, similar to findings in the literature [43]. To delve deeper into carrier recombination dynamics, TRPL tests were conducted and are depicted in Fig. 1f. The TRPL data exhibited a complex decay behavior, which could be described by two exponentials with characteristic times of approximately 6 and 286 ns, respectively. The shorter decay time likely corresponds to the interband recombination of electrons and holes in the direct-gap semiconductor, consistent with similar observations in previous studies [44]. On the other hand, the longer decay time may suggest the influence of trapping levels and defect states on the recombination dynamics. It is plausible that nonequilibrium carriers are captured at these levels, which lie near the band minima, and subsequently released into allowed bands with subsequent recombination [44]. These TRPL findings reveal an extended carrier lifetime in the perovskite films, suggesting the potential for constructing high-efficiency photovoltaic devices.

**Fig. 2:**
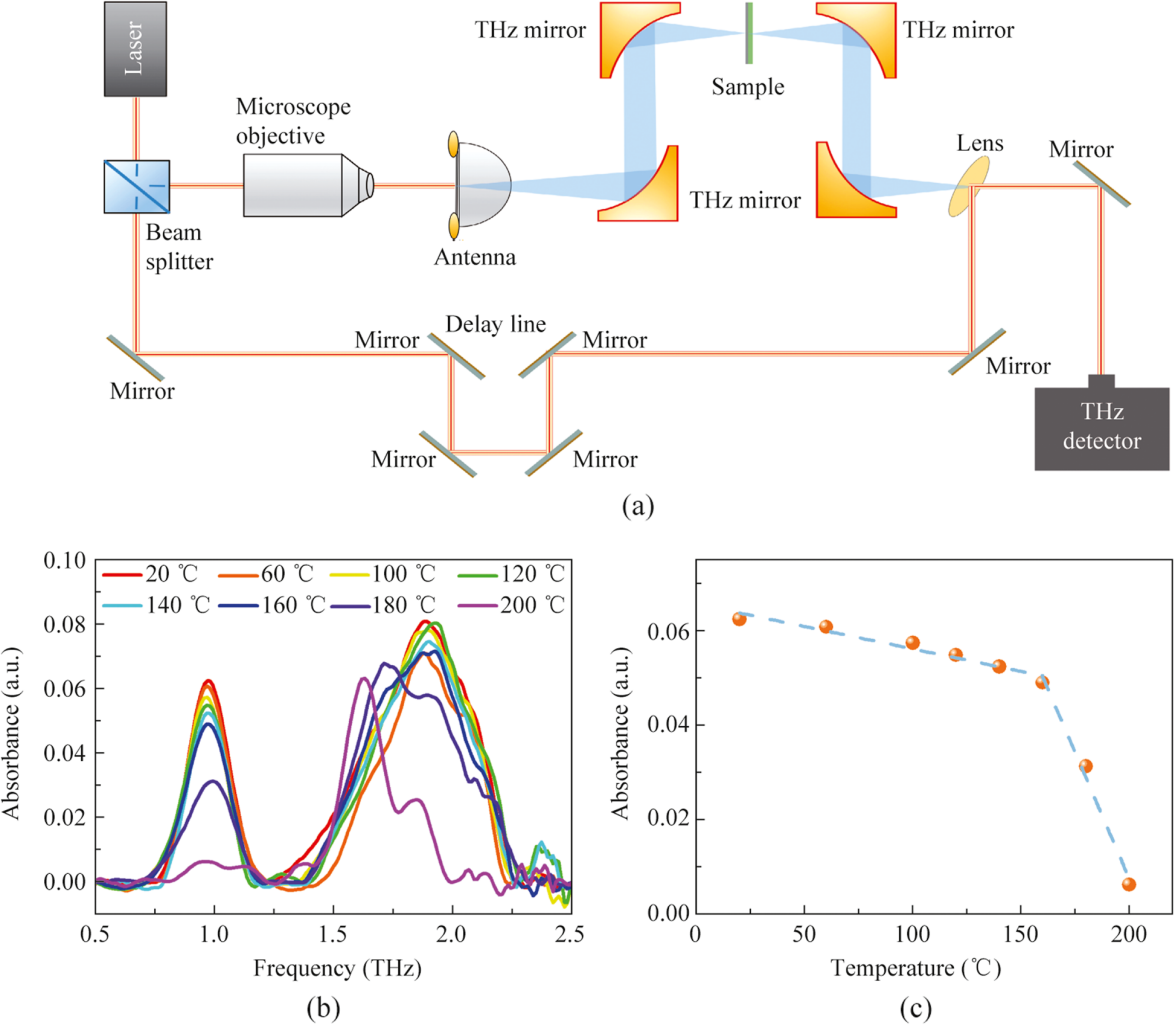
**a** Schematic diagram of the THz-TDS system. **b** Terahertz absorption spectra of the MAPbI_3_ films aged at different temperatures. **c** Intensity of absorption peak at 0.968 THz aged at different temperatures

After employing the traditional characterization methods mentioned above to analyze the quality of the synthesized perovskite films, we systematically characterized the perovskite films at different aging status using a THz-TDS system. Figure 2a depicts the intricate configuration of the classical THz-TDS system, comprising essential components like a precise femtosecond laser, an antenna, a delay line, a sensitive terahertz detector, and optical elements. The femtosecond laser pulse is split into two beams using a beam splitter: one as excitation light to activate the terahertz source, generating terahertz radiation, and the other, after passing through a delay line, focuses on the terahertz detector for beam detection. The THz-TDS system provides a versatile platform for material non-destructive detection, including precise determination of the terahertz absorption coefficient and measurement of other parameters like the dielectric constant, offering valuable insights into electromagnetic properties in the terahertz frequency range. The observed absorption peaks in the terahertz frequency range of semiconductors are commonly attributed to lattice vibrations. When the frequency of lattice vibrations coincides with that of the incident terahertz wave, a resonance occurs, resulting in the absorption of terahertz energy and the emergence of distinct absorption peaks at corresponding frequencies. By analyzing the intensity and position of these absorption peaks, changes in the lattice structure of the material can be inferred. In this investigation, the organic–inorganic hybrid perovskite experiences a gradual deterioration of its lattice structure as the aging process advances, potentially causing alterations in the intensity or position of the terahertz absorption peaks. Therefore, by analyzing the terahertz absorption peaks in perovskite samples at different degradation status, a quantifiable measure of the perovskite’s degradation degree can be obtained.

It is worth noting that the terahertz time-domain spectroscopy system provides time-domain spectroscopic data of the samples. To obtain the absorption data of the samples, processing of the time-domain spectroscopic data are required. In this study, we employed a transmission mode for sample characterization. To acquire the absorbance spectra of the samples, the time-domain spectroscopic data were Fourier transformed to produce frequency-domain spectra, and subsequently, the absorbance (*A*(*v*)) of the samples can be obtained based on the following equation [45, 46].

$$A\left(v\right)=\text{log}\left(\frac{{I}_{\text{ref}}\left(v\right)}{{I}_{\text{sam}}\left(v\right)}\right),$$

where *I*_ref_(*v*) represents the intensity of the reference sample, and *I*_sam_(*v*) represents the intensity of the test sample.

As shown in Fig. 2b, the terahertz absorption spectra of MAPbI_3_ thin films were subjected to analysis, which unveiled the presence of two distinct absorption peaks at 0.968 and 1.895 THz. This is consistent with previous research results, both theoretical [47] and experimental [38, 39, 48], which indicate that the absorption peak at 0.968 THz is caused by the buckling of the I-Pb-I bond angles, while the peak at 1.895 THz is due to the Pb-I length vibrations. Notably, as the aging level of the perovskite increases, particularly under elevated aging temperatures, the absorption intensity of the characteristic peak at 0.968 THz gradually diminishes, accompanied by a slight blue shift in the peak position. In contrast, the absorption peak intensity at 1.895 THz does not exhibit a monotonic relationship with the aging temperature, but instead demonstrates a perceptible red shift in the peak position. Numerous studies about MAPbI_3_ aging have revealed that the degradation of perovskite is influenced by various factors, and the decay process of perovskite crystals is highly intricate [49–52]. The detected irregular displacements in the peak position of the terahertz absorption peak at 1.895 THz, observed under varying aging conditions in this study, further suggesting the presence of complex aging processes in perovskite crystals.

The analysis of terahertz absorption peaks facilitates a quantitative evaluation of the aging level and structural changes occurring in perovskite materials, thereby providing valuable guidance for their application and performance optimization. Therefore, the peak intensity of the absorption peak at 0.968 THz may serve as a quantitative indicator for evaluating the degradation status of perovskite films. Figure 2c illustrates the variation of this absorption peak (0.968 THz) intensity with aging temperature. It can be observed that the absorption peak intensity exhibits a slight attenuation of approximately 20% as aging temperature increases from 20 °C to 160 °C. However, once the aging temperature exceeds 160 °C, the absorption peak intensity decreases rapidly from 0.049 to 0.006, recorded a peak intensity reduce of about 90%. Given that the absorption peak at 0.968 THz corresponds to the bending of the I-Pb-I bonds, this observation suggests that the perovskite demonstrates enhanced stability at temperatures below 160 °C. However, as the aging temperatures exceed 160 °C, the integrity of its lattice framework gradually declines, accelerating the degradation of the perovskite crystal. Besides, it is important to note that the absorption peak intensity demonstrates linear decay characteristics in both the low-temperature region below 160 °C and the high-temperature region above 160 °C. Therefore, employing this absorption peak intensity as a quantitative metric enables accurate assessment of the degradation status of MAPbI_3_ films.

**Fig. 3:**
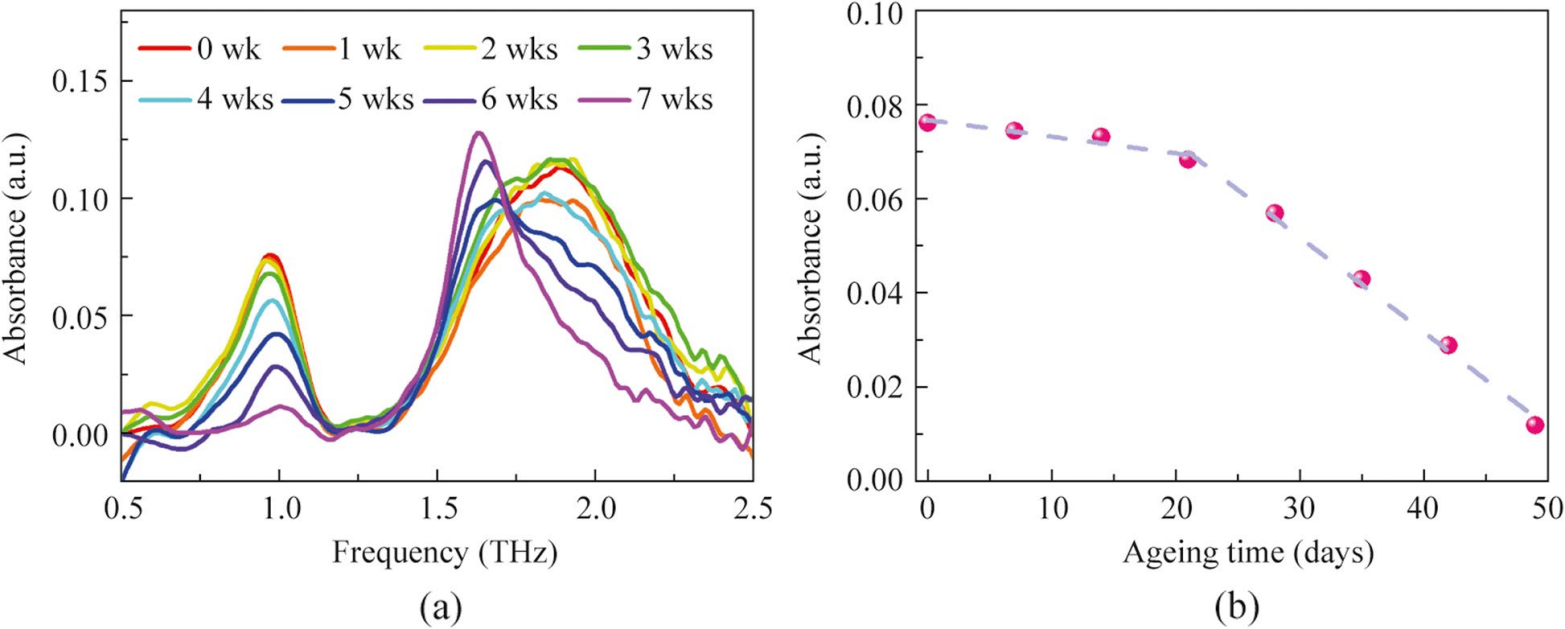
**a** Terahertz absorption spectra of the MAPbI_3_ films aged at room temperature and in atmosphere. **b** Intensity of absorption peak at 0.968 THz aged in ambient conditions

To further investigate the aging behavior of perovskite films in a typical operational environment, we conducted natural aging experiments of perovskite films under ambient atmospheric conditions. As shown in Fig. 3a, during a seven-week natural aging experiment, the intensity of the characteristic absorption peak at 0.968 THz decreased with increasing aging time, consistent with the results obtained from the aforementioned thermal decomposition aging experiment. This finding indicates that perovskite exhibits a similar aging trend through room temperature natural aging as it does through thermal decomposition. It is noteworthy that, while environmental conditions such as temperature, humidity, and oxygen concentration remained constant throughout the entire aging process, the aging rate of perovskite films was not consistent. As shown in Fig. 3b, the perovskite aging process proceeded at a slower pace during the initial three weeks, but beyond three weeks, there was a pronounced acceleration in the aging rate of perovskite films. The underlying mechanisms for this abnormal aging behavior remain unclear and warrant further investigation in future studies.

**Fig. 4:**
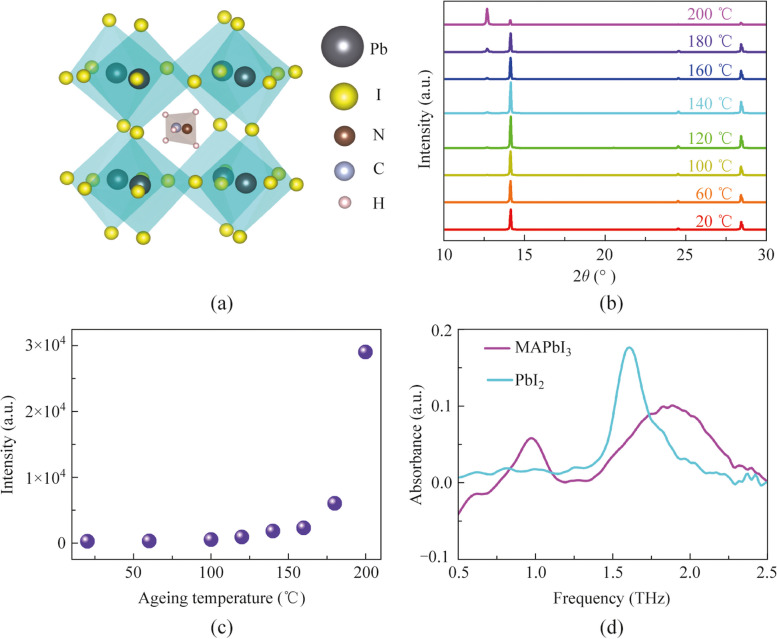
**a** Schematic diagram of the crystal structure of MAPbI_3_. **b** XRD spectra of the perovskite films aged at different temperatures from 20 °C to 200 °C. **c** XRD peak intensity of PbI_2_ (2*θ* = 12.7°) in MAPbI_3_ films aged at different temperatures. **d** Terahertz absorption spectra of MAPbI_3_ and PbI_2_ films

Although it is known that the terahertz absorption peaks at 0.968 and 1.895 THz correspond to the buckling of the I-Pb-I bond angles and the stretching vibration of the Pb-I bond, respectively, the underlying reasons for the changes in the intensity and position of these peaks during the aging of perovskite are not fully understood. To gain deeper insights, we systematically analyzed the aging process from various perspectives, including the structure of perovskite, the aging process, the terahertz absorption spectra of the raw materials used to synthesize perovskite, and the XRD spectra of perovskite at different aging stages. As shown in Fig. 4a, the methylammonium ion, situated within a cage formed by four PbI_6_ octahedra, exhibits conditional mobility within this confined structure [40]. The aging of perovskite involves the formation of defects, oxidation, distortion of the crystal structure, and eventual decomposition of the crystal. The octahedral framework of perovskite is soft due to the random orientation of the internal organic groups [53, 54]. During the aging process, this soft structure can lead to distortions in the I-Pb-I bond angles, potentially altering its vibrational frequency. This may explain the slight blue shift of the absorption peak at 0.968 THz as aging progresses. Figure 4b presents the XRD spectra of perovskite at different aging stages. The unaged perovskite shows three main peaks corresponding to the (110), (202), and (220) planes. When the aging temperature reaches 120 °C, the XRD spectrum reveals the (001) peak of PbI_2_ at 12.7° (2*θ*) (JCPDS No. 00-007-0235), indicating the formation of CH_3_NH_2_ defects and PbI_2_ crystals. As the aging temperature increases, the intensity of the PbI_2_ peak continues to rise (Fig. 4c). Below 160 °C, the increase in PbI_2_ content is gradual, but above 160 °C, it rises sharply, mirroring the red shift trend of the terahertz absorption peak at 1.895 THz. We hypothesize that the red shift of the 1.895 THz absorption peak is due to the formation of PbI_2_. To verify this, we measured the terahertz absorption spectrum of PbI_2_, which shows an absorption peak at 1.617 THz (Fig. 4d). This peak is close to the position of the absorption peak in perovskite aged at 200 °C and after 7 weeks of natural aging, indicating that the final aging product is PbI_2_, consistent with the XRD results. This supports the idea that the red shift at 1.895 THz is due to PbI_2_ absorption. Additionally, during the aging process, the intensity of the 0.968 THz peak decreases with increasing aging. From the XRD spectra at different aging temperatures, it is evident that the PbI_2_ content increases rapidly above 160 °C, suggesting that the crystal structure of perovskite is disintegrating rapidly. This rapid disintegration could explain the swift attenuation of the absorption peak at 0.968 THz. Below 160 °C, the absorption peak’s intensity decreases slowly, likely due to defect formation disrupting the regular lattice structure, affecting the I-Pb-I bond vibration. Besides, increased defect density absorbs or scatters the energy contributing to the terahertz absorption peak, weakening the signal. Interestingly, during natural aging, the intensity of the peak at 1.895 THz increases after more than 5 weeks. Comparing the absorption peak intensities of perovskite and PbI_2_ (Fig. 4d), we find that PbI_2_’s absorption peak at 1.617 THz is significantly stronger than that of perovskite at 1.895 THz. This may be because the electronic structure of PbI_2_ facilitates strong terahertz absorption. PbI_2_ has a direct bandgap and strong excitonic effects, enhancing the coupling between electronic states and lattice vibrations, leading to stronger absorption features. Therefore, we suggest that as perovskite undergoes natural aging and its crystal structure disintegrates, the increased formation of PbI_2_ leads to a gradual increase in the absorption peak’s intensity due to PbI_2_’s strong terahertz absorption properties. These discoveries imply the potential for terahertz spectral techniques to accurately assess the aging status of organic–inorganic hybrid perovskites in real time and continuously monitor the performance and degradation of perovskite optoelectronic devices.

## Conclusions

In conclusion, the terahertz absorption spectra of MAPbI_3_ thin films with different degradation degrees, which aged by thermal decomposition and the natural aging method in ambient environment, were investigated. The results revealed two distinct absorption peaks in the 0.5 to 2.5 THz range for the as-prepared perovskite films. The absorption peak at 0.968 THz exhibited a gradual decrease in intensity and a slight blue shift in position with increasing aging. Meanwhile, the absorption peak at 1.895 THz showed a red shift in peak position as the perovskite aged. By combining the crystal structure of perovskites, XRD spectra at different aging stages, and their aging process, we provide an explanation for the changes in intensity and peak position of the two terahertz absorption peaks as the perovskite aged. The results indicate that the blue shift of the absorption peak at 0.968 THz is likely due to the distortion of the I-Pb-I bond angles caused by crystal aging, while the attenuation of its intensity is associated with an increase in crystal defects and the disruption of the I-Pb-I bond angles. Meanwhile, the red shift of the peak at 1.895 THz is attributed to the formation of PbI_2_ during the aging process. The present study holds promising potential for quantifying and monitoring the aging level of organic–inorganic hybrid perovskite in real-time. These findings may contribute to the fundamental research of perovskites and advancing the industrial utilization of perovskite materials.

## Data Availability

The data that support the findings of this study are available from the corresponding author, upon reasonable request.
